# Optimizing gentamicin conventional and extended interval dosing in neonates using Monte Carlo simulation – a retrospective study

**DOI:** 10.1186/s12887-019-1676-3

**Published:** 2019-09-06

**Authors:** Monique Bergenwall, Sandra A. N. Walker, Marion Elligsen, Dolores C. Iaboni, Carla Findlater, Winnie Seto, Eugene Ng

**Affiliations:** 1 Department of Pharmacy, Sunnybrook Health Sciences Centre, 2075 Bayview Avenue, E-302, Toronto, ON M4N 3M5 Canada; 2 Leslie L. Dan Faculty of Pharmacy, University of Toronto, Toronto, ON Canada; 3 Division of Infectious Diseases, Sunnybrook Health Sciences Centre, Toronto, ON Canada; 4 Sunnybrook Health Sciences Centre Research Institute, Toronto, ON Canada; 5 Women and Babies Program, Sunnybrook Health Sciences Centre, Toronto, ON Canada; 6 Department of Pharmacy, Hospital for Sick Children, Toronto, ON Canada; 7 Department of Paediatrics, University of Toronto, Toronto, ON Canada; 8 Present Address: Grandview Medical Centre Family Health Team, 167 Hespeler Rd, Cambridge, ON N1R 3H7 Canada

**Keywords:** Neonate, Gentamicin, Pharmacokinetics, Traditional dosing, extended-interval dosing, Monte Carlo simulation

## Abstract

**Background:**

Although aminoglycosides are routinely used in neonates, controversy exists regarding empiric dosing regimens. The objectives were to determine gentamicin pharmacokinetics in neonates, and develop initial mg/kg dosing recommendations that optimized target peak and trough concentration attainment for conventional and extended-interval dosing (EID) regimens.

**Methods:**

Patient demographics and steady-state gentamicin concentration data were retrospectively collected for 60 neonates with no renal impairment admitted to a level III neonatal intensive care unit. Mean pharmacokinetics were calculated and multiple linear regression was performed to determine significant covariates of clearance (L/h) and volume of distribution (L). Classification and regression tree (CART) analysis identified breakpoints for significant covariates. Monte Carlo Simulation (MCS) was used to determine optimal dosing recommendations for each CART-identified sub-group.

**Results:**

Gentamicin clearance and volume of distribution were significantly associated with weight at gentamicin initiation. CART-identified breakpoints for weight at gentamicin initiation were: ≤ 850 g, 851-1200 g, and > 1200 g. MCS identified that a conventional dose of gentamicin 3.5 mg/kg given every 48 h or an EID of 8-9 mg/kg administered every 72 h in neonates weighing ≤ 850 g, and every 24 and 48 h, respectively, in neonates weighing 851-1200 g, provided the best probability of attaining conventional (peak: 5-10 mg/L and trough: ≤ 2 mg/L) and EID targets (peak:12-20 mg/L, trough:≤ 0.5 mg/L). Insufficient sample size in the > 1200 g neonatal group precluded further investigation of this weight category.

**Conclusions:**

This study provides initial gentamicin dosing recommendations that optimize target attainment for conventional and EID regimens in neonates weighing ≤ 1200 g. Prospective validation and empiric dose optimization for neonates > 1200 g is needed.

## Background

Although aminoglycosides are routinely used in neonates, controversy exists regarding recommended empiric dosing to optimize target attainment with either conventional dosing (peak: 5-10 mg/L and troughs ≤2 mg/L) or extended-interval dosing (higher peak and undetectable trough) [1, 2]. In adult and older pediatric populations, EID regimens targeting peak concentrations of ≥ 20 mg/L are routinely recommended based on data suggesting that aminoglycoside activity is optimized with peak: minimum inhibitory concentration (MIC) ratios of 8–10:1 [3–5]. For these patient populations, EID has consistently demonstrated equal efficacy, and equal or reduced toxicity versus conventional dosing [1, 6–10].

While data exist to support the use of EID in neonates [6, 11–26], consensus is lacking regarding optimal EID target concentrations that optimize efficacy and minimize toxicity in this patient population. Peak concentrations investigated in neonates vary from 4 to 20 mg/L [15–26], and typically remain below 12 mg/L, with no clear rationale. Furthermore, infants born at a gestational age (GA) ≤ 28 weeks, along with those with a birth weight (BW) of ≤1500 g, are underrepresented in EID studies. These infants constitute approximately 20% of all neonates admitted to Canadian neonatal intensive care units (NICUs), and 50% of those admitted to Level III NICUs [27]. Since aminoglycoside pharmacokinetic (PK) parameters in neonates may be influenced by weight [15, 18–20, 28, 29], gestational age [15, 28, 29] and postnatal age [19, 28, 29], further research is required in this unique population in order to optimize target attainment and thereby, maximize the probability of efficacy of the antibiotic while minimizing the risk of nephrotoxicity.

The objectives of this study were to determine the pharmacokinetics of gentamicin in neonates with no clinical evidence of renal impairment in a Level III NICU, identify significant covariates of gentamicin PK parameters in neonates, and develop practical initial dosing recommendations with the highest probability of attaining target peak and trough serum concentrations currently accepted in clinical practice for both conventional dosing (trough < 2 mg/L and peak 5–10) and EID (trough < 0.5 mg/L and peak 8-20 mg/L, 12-20 mg/L, 15-20 mg/L and > 20 mg/L) of gentamicin.

## Methods

This retrospective study was conducted in the level III NICU at Sunnybrook Health Sciences Centre (SHSC) in Toronto, Ontario, Canada. SHSC is a 1325-bed tertiary care teaching hospital, with 48 NICU beds [30].

### Patient eligibility

Neonates admitted to the NICU from March 12th, 2010-November 26th, 2013 who were prescribed gentamicin to treat a documented or presumed infection and received > 48 h of gentamicin were identified from a hospital electronic database [31]. Patients with at least one set of steady state gentamicin serum concentrations (trough and peak concentrations obtained at the earliest before and after the third dose of a given dosing regimen, respectively) with documentation of gentamicin administration and serum sampling times were included.

Neonates were excluded if they developed acute renal failure (urine output < 1 mL/kg/hr. or serum creatinine [sCr] > 100 μmol/L) before or during gentamicin therapy, had an increase in sCr > 25% from baseline during treatment, or had a calculated gentamicin half-life > two standard deviations (SDs) from the mean half-life observed in the study population following data analysis, without the availability of an additional set of serum concentrations to confirm the accuracy of this calculated half-life.

### Gentamicin dosing and sampling procedure

At the time of this study, neonatal SHSC conventional gentamicin dosing recommendations aimed to target a peak and trough serum concentration of 5–10 mg/L and ≤ 2 mg/L, respectively. (Appendix 1).

### Gentamicin pharmacokinetics

The PK profile of gentamicin in neonates has been previously described using one [14, 15, 17–19, 21, 24], two [20, 25, 29] and three [28] compartment models. Once gentamicin distribution is complete, it follows first order elimination [15, 20, 28]. Therefore, a one compartment model is appropriate to evaluate the post-distribution pharmacokinetics of gentamicin. Gentamicin concentrations were analyzed using first order PK principles to calculate extrapolated gentamicin trough and peak, elimination rate constant (k_e_), half-life (t_1/2_), volume of distribution (Vd), clearance (Cl), initial estimated dose (mg/kg, rounded to nearest 0.5 mg) and dosing interval for conventional (trough ≤ 2 mg/L and peak 5-10 mg/L) and EID (trough ≤ 0.5 mg/L and peak 8-20 mg/L, 12-20 mg/L, 15-20 mg/L and > 20 mg/L) using an infusion time of 1 h. (Appendix 2) When multiple sets of gentamicin serum concentrations were obtained from the same patient, each set was evaluated independently for inclusion, and if eligible, was included as a separate sample for the PK analysis along with the corresponding post-natal age (PNA) and corrected GA (CGA) at time of gentamicin initiation; weight closest to gentamicin initiation; and weight within 24 h of gentamicin levels.

### Microbiological cultures

Data for all positive bacterial isolates along with the culture source were extracted from the hospital electronic data base and patient charts.

### Statistical analysis

Descriptive statistics were used for patient characteristics and microbiological results (number, percent, mean, SD and range). Since PK parameters display a lognormal distribution, the geometric mean, 95% confidence interval (CI) and range were reported for k_e_, t_1/2_, Vd, and Cl.

The data consisted of 60 neonates, of which only 4 had a second set of data with gentamicin levels. This sample size, along with the limited number of repeated measures, was insufficient to run a robust hierarchical model. To circumvent this problem, only data from the first set of gentamicin levels were included for the analyses. Clinical parameters that would have been known prior to the initiation of gentamicin, were not calculated using other parameters input into the regression analysis and were parameters with values available for > 80% of the gentamicin levels (GA at birth; CGA at gentamicin initiation; PNA at gentamicin initiation; gender; BW; weight at gentamicin initiation; Apgar score at one and 5 min of age; blood urea nitrogen [BUN] closest to gentamicin initiation, sCr closest to gentamicin initiation, 24 h urine output [ml/hr], and albumin closest to gentamicin initiation; use of concomitant nephrotoxins [indomethacin, ibuprofen, furosemide, amphotericin B, vancomycin]; and small-for-gestational age [SGA; *i.e* neonates with a birth weight below the 10th percentile for neonates of the same GA] status) were input in the regression analysis. Variables that were significant (*p* <  0.05) with bivariate analysis and had a tolerance statistic of ≥0.4 when assessed for multicollinearity were included in a multivariable linear regression (MLR) model to identify those that remained significant using a *p* <  0.05. Analyses were run using SAS Version 9.4 (SAS Institute, Cary, NC, USA).

A Classification and Regression Tree (CART) analysis (CART1 Professional Extended Edition, Salford Systems, San Diego, California) was used to identify whether practical breakpoints existed for statistically significant MLR-identified covariates of gentamicin Cl (L/h) and Vd (L). The initial CART analyses input all statistically significant variables identified in the MLR analyses for Vd (L) and/or Cl (L/h). CART analyses for Cl and Vd were pruned to the simplest tree, utilizing forced splits to identify clinically practical breakpoints, with the lowest relative error. Forced splits were selected as practical rounded breakpoints derived from the CART identified breakpoint and which had equal or lower relative error than the CART identified breakpoint. The optimal CART model was that which allowed for the fewest sub-groups and had the lowest relative error. CART-identified breakpoints for covariates of gentamicin Vd and/or Cl were used to create patient sub-groups. Mean pharmacokinetic data were calculated for each identified sub-group and the sub-groups were compared to verify the existence of a significant difference in pharmacokinetic parameters (ke [h^− 1^], Vd [L/kg], and Cl [L/h/kg]) to confirm the validity of the CART-identified breakpoints. An analysis of variance (ANOVA) with Tukey-Kramer Multiple Comparisons Test for data that passed the test for normality or a Kruskal-Wallis Test with Dunn’s Multiple Comparison Test for data that did not pass the test for normality (if > 2 sub-groups were identified) or unpaired t-test (if 2 sub-groups were identified) were used to compare the sub-groups (GraphPad InStat version 3.05, 32-bit for Win95/NT; GraphPad Software Inc., La Jolla, California) for differences in pharmacokinetic parameters with a *p* <  0.05 considered statistically significant.

Mean PK data of each sub-group were used to explore initial dosing recommendations using first order PK equations for a suggested dose and interval based on inputs for the desired peak and trough concentrations with an infusion time of 1 h. The exploratory gentamicin dose and intervals were subsequently evaluated using Monte Carlo simulation (Oracle Crystal Ball, version 11.1.2.4.000, 32-bit for Windows, Redwood City, California) (MCS). The mean and SDs for k_e_, Vd, and weight for each determined patient subgroup were input with one million iterations to determine the probability of attaining target steady state peak gentamicin concentrations of 5–10 mg/L, 8–12 mg/L, 8–15 mg/L, 8–20 mg/L, 12–20 mg/L, 15–20 mg/L and > 20 mg/L, as well as target trough concentrations of ≤2 mg/L and ≤ 0.5 mg/L with any given dosing simulation. For the purpose of the MCSs, k_e_ and Vd were assigned a lognormal distribution; weight was assumed to have a triangular distribution and was truncated at the value corresponding to the CART analysis breakpoint for weight for the given sub-group. The upper and lower limits for weight selection were truncated at 4 kg and 0.3 kg, respectively, to reflect values above and below which would be improbable for surviving neonates (< 0.3 kg) and would be greater than 2 SDs from the mean of any sub-group weight category. As part of each MCS, an assessment of the probability of attaining a Peak:MIC ratio of ≥8 was completed. The MIC was assumed to have a normal distribution truncated at a minimum of 0.5 mg/L and maximum of 8 mg/L (Clinical and Laboratory Standards Institute breakpoint for intermediate susceptibility of Enterobactereaceae to gentamicin [32]) with a mean MIC_90_ of 2 mg/L and SD of 1 mg/L, resembling the current MIC distribution for *E coli* in Canadian pediatric patients [33].

## Results

### Demographics

**Fig. 1:**
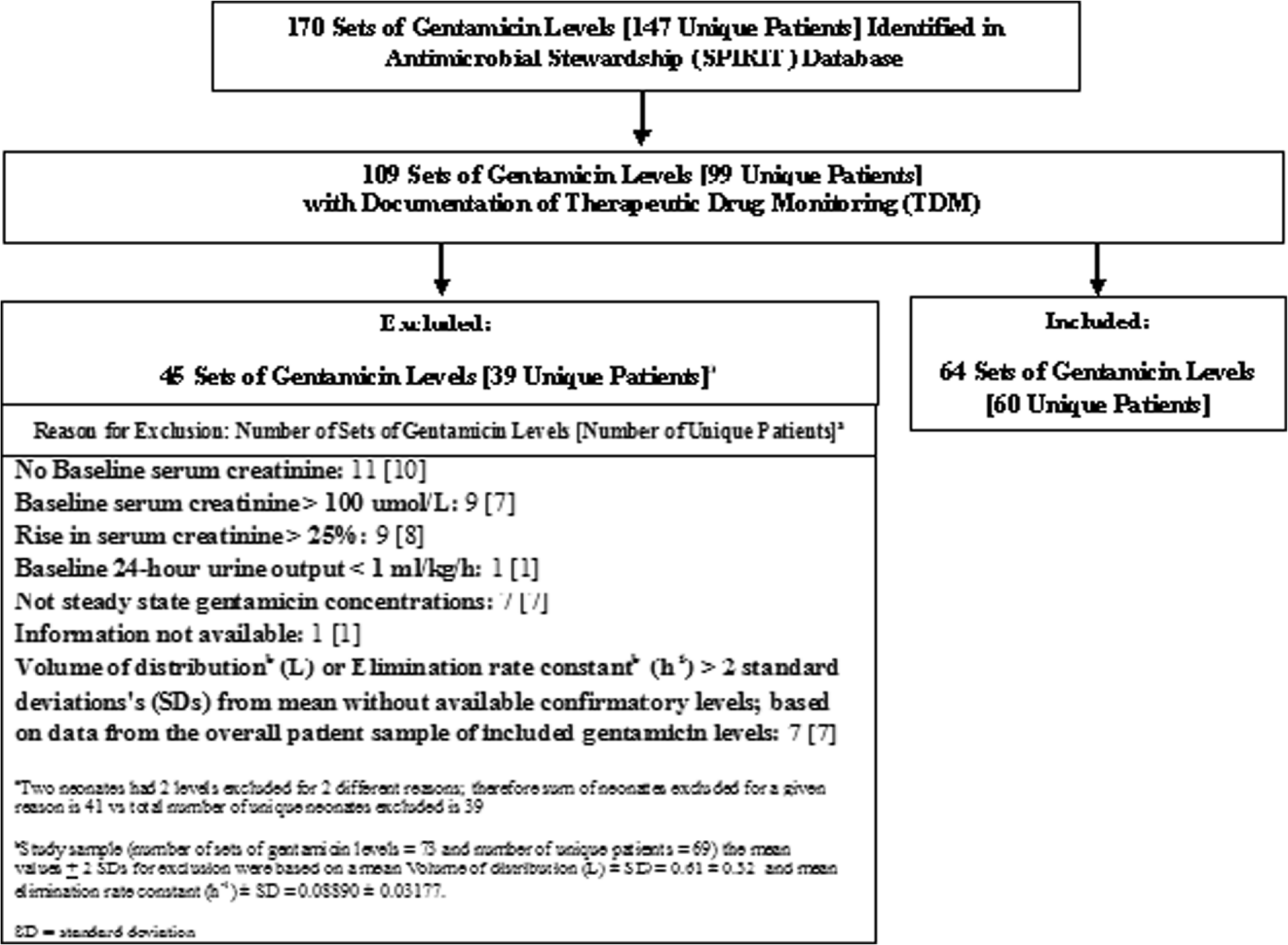
Study Eligibility

Of a total of 99 patients for whom there was documentation of therapeutic drug monitoring (TDM), 60 patients were eligible for study inclusion to complete the pharmacokinetic analysis (Fig. 1 and Table 1). Patients with a rise in sCr of > 25% during gentamicin therapy were excluded and represent patients who developed nephrotoxicity while on gentamicin (8/99 patients (8%)); recognizing that nephrotoxicity may have been multifactorial and no assumptions can be made about causation associated with gentamicin in this retrospective study (Fig. 1).

**Table 1:** Patient Characteristics

Patient Demographics Based on Number of Patients = 60	Mean ± Standard Deviation (Range)	Number (%)
Gender (Female)		24 (40)
Gestational Age at Birth (Weeks)	27 ± 3 (23–36)	
Birth Weight (g)	990 ± 482 (488–2740)	
Small for Gestational Age Status (Yes)		1 (2)
Place of Birth (Outborn)		15 (25)
Apgar Score at 1 minute^a^	5 (1–9)	
Apgar Score at 5 minute^a^	7 (2–9)	
Neonatal Intensive Care Unit Survival		56 (93)
Gentamicin Treatment (Based on Number of Sets of Gentamicin Levels = 64)		
Post-Natal Age at Gentamicin Initiation (Days)	10 ± 12 (1–46)	
Corrected Gestational Age at Gentamicin Initiation (Weeks)	28 ± 3 (24–36)	
Weight at Gentamicin Initiation(g)	1059 ± 496 (488–2789)	
Gentamicin Dose (mg/kg/dose)	3.0 ± 0.7 (2–5.6)	
Gentamicin Dosing Interval (Hours)^a^	24 (12–36)	
Duration of Gentamicin Therapy (Days)	7 ± 2 (2–13)	
Indication for Antibiotic Therapy^b^ (Based on Number of Patients = 60)		
Culture Negative Sepsis		30 (50)
Necrotizing enterocolitis Septic Ileus		10 (17)
Respiratory Tract Infection		6 (10)
Empiric Treatment (< 5 days)		5 (8)
Confirmed Sepsis		4 (7)
Urinary Tract Infection		4 (7)
Meningitis		2 (3)
Skin and Soft Tissue Infection		1 (2)
Other		1 (2)
Laboratory Parameters (Closest to and BEFORE Gentamicin Start Date, unless otherwise noted) (Based on Number of Sets of Gentamicin Levels = 64)		
Serum creatinine (μmol/L)	57 ± 21 (24–93)	
Maximum serum creatinine during Gentamicin (μmol/L)	64 + 23 (19–100)	
Blood Urea Nitrogen (mmol/L)	8 ± 5 (2–24)	
Maximum blood urea nitrogen during Gentamicin (mmol/L)	11 ± 5 (3–26)	
24-h urine output (ml/kg/hr)	4 ± 1 (1–7)	
Lowest 24-h urine output during Gentamicin (ml/kg/hr)	3 ± 2 (1–19)	
Trough gentamicin concentration (mg/L)^c^	1.1 ± 0.6 (0.2–3.9)	
Peak gentamicin concentration (mg/L)^c^	7.1 ± 2.2 (3.7–17.1)	
Nephrotoxins & Ototoxins^d^ (Based on Number of Sets of Gentamicin Levels = 64)		
Concomitant Nephrotoxins during a course of gentamicin		43 (67)
Concomitant Ototoxins during a course of gentamicin		44 (69)
Vancomycin^e,f^		25 (39)
Indomethacin^e,f^		21 (33)
Furosemide^e,f^		3 (5)
Amphotericin B^e^		3 (5)
Ibuprofen^e,f^		1 (2)
Erythromycin^f^		2 (3)
Prior course of Gentamicin		30 (47)

^a^Median reported since apgar scores are ordinal data and standard dosing gentamicin intervals were used (e.g. every 12, 24, or 36 h), therefore, gentamicin dosing interval data are ordinal

^b^Three patients with 2 sets of gentamicin levels had a different diagnosis for each set of gentamicin levels. Therefore, the sum (%) of total indications is greater than 60 (100%) (i.e. 63 (105%))

^c^Extrapolated concentration using first order pharmacokinetics

^d^Each course of gentamicin may have had greater than one nephrotoxin or ototoxin, therefore, sum of individual nephrotoxins and ototoxins is greater than the total number of courses of gentamicin with a concomitant nephrotoxin or ototoxin

^e^Nephrotoxin

^f^Ototoxin

Forty-five of the 60 neonates (75%) included in this study were born at ≤28 weeks gestation. The mean (± standard deviation (SD), range) GA of neonates at birth and CGA at gentamicin initiation were 27 (± 3, 23–36) weeks and 28 (± 3, 24–36) weeks, respectively. Thirty-nine patients (65%) had a BW of < 1000 g (defined as extremely low BW [34]) and 55 patients (92%) had a BW of < 1500 g (defined as very low BW [34]). In this cohort, gentamicin was most commonly used for the treatment of culture negative sepsis (30/60; 50%). Forty-four percent (16/36) of all bacterial isolates were gram-negative bacteria (GNB), most commonly *Escherichia coli* (7/36; 19%) and *Klebsiella spp (*5/36; 14%) (Table 2).

**Table 2:** Bacterial Isolates Cultured at Time of Gentamicin Initiation

Number of gentamicin treatment courses		64 (4 patients had 2 separate gentamicin treatment courses)					
Number of gentamicin treatment courses with a positive culture (%)		24 (37.5)					
Number of gentamicin treatment courses that were Polymicrobial (2 or more bacterial isolates) (%)		7 (10.9)					
	Total Number of Isolates *n* = 36 (%)^a^	Source of Culture					
		Blood	Cerebrospinal Fluid	Endotracheal Tube	Urine	Eye	Skin
*Escherichia coli*	7 (19)	4 (11)	1 (3)	1 (3)	1 (3)	0	0
*Klebsiella* species	5 (14)	1 (3)	0	2 (6)	2 (6)	0	0
*Pseudomonas* species	2 (6)	0	0	1 (3)	0	1 (3)	0
*Enterobacter* species	1 (3)	0	0	0	0	0	1 (3)
*Raoultella* species	1 (3)	0	0	0	0	0	1 (3)
Gram-positive organisms^b^	15 (42)	7 (19)	1 (3)	1 (3)	3 (9)	1 (3)	2 (6)
Other^c^	5 (14)	0	0	5 (14)	0	0	0

^a^ All percentages are determined from total isolates (*n* = 36)

^b^ Total of 15 g positive organisms include coagulase-negative *Staphylococcus* (12); *Enterococcus* species (1); *Staphylococcus aureus* (1) Group B *Streptococcus* (1)

^c^Total of 5 ‘Other’ organisms include *Mycoplasma spp* (1) and *Ureaplasma urealyticum* (4)

### Bivariate and multivariable analyses

Significant predictors (*p* < 0.05) of gentamicin Vd (L) and Cl (L/h) from the bivariate screen and multivariable model are detailed in Table 3. The only covariate that remained significant following MLR for Vd (L) was weight at gentamicin initiation (*P* < 0.0001). Covariates that remained significant following MLR for Cl (L/h) were PNA at gentamicin initiation (*p* = 0.0001), gender (*p* = 0.0447), and weight at gentamicin initiation (*p* < 0.0001).

**Table 3:** Bivariate and Multivariable Analysis

Parameter^a^	Clearance (L/h)		Volume of Distribution (L)	
	Bivariate *p*-value	Multivariable *p*-value	Bivariate *p*-value	Multivariable *p*-value
Post-natal age (Days) at gentamicin initiation	**< 0.0001**	**0.0001**	**0.0037**	0.0563
Gender	**0.0311**	**0.0447**	0.0667	–
Weight at gentamicin initiation (g)	**< 0.0001**	**< 0.0001**	**< 0.0001**	**< 0.0001**
Blood urea nitrogen at baseline (mmol/L)	**< 0.0001**	0.5855	**< 0.0001**	0.6643
Serum creatinine at baseline (μmol/L)	**< 0.0001**	0.0569	**0.0011**	0.4553
Concomitant nephrotoxins	**0.0154**	0.9332	**0.0279**	0.8204

^a^Baseline values needed to be reported within 14 days prior to the initiation of gentamicin; if unavailable, first value taken during course of gentamicin was used as a surrogate

Bold data indicates statistically significant *p*-values for a given parameter with either bivariate or multivariable analysis

### CART analysis

The optimal CART analyses for Vd(L) and Cl(L/h) produced breakpoints based on the patients’ weight at gentamicin initiation, with a forced split at ≤ 850 g, > 850 g – 1200 g, and > 1200 g. These breakpoints provided the simplest trees with the lowest relative error (Relative Error for Vd tree = 0.347; Relative Error for Cl tree = 0.344). CART identified trees and breakpoints for other parameters in the MLR regression equations (PNA and gender) did not exist.

The mean k_e_ and Cl (L/h/kg) for neonates ≤ 850 g were significantly different from the other weight breakpoints (Table 4). Mean pharmacokinetic parameters for neonates weighing 851 - 1200 g versus > 1200 g were not statistically different (*p* > 0.05) (Table 4). The small number of participants (*n* = 13, with 15 gentamicin levels), limited weight range (1210-2789 g; mean 1744 g) and wide confidence intervals of the mean calculated pharmacokinetic parameters in the > 1200 g weight sub-group caused concern regarding the robustness of any dosing recommendations derived for this weight sub-category. As a result, the > 1200 g weight sub-category of neonates was excluded from further analyses. The significant difference in both k_e_ and Cl (L/h/kg) between the ≤850 g and 851-1200 g sub-groups (Table 4), and absence of CART identified trees and breakpoints for the other MLR equation covariates (PNA and gender) supports the use of the simple weight range breakpoints of ≤850 g and 851-1200 g as the sub-groups for practical and convenient empiric gentamicin dosing calculations in neonates.

**Table 4:** Mean pharmacokinetic parameters

	≤ 850 g			851–1200 g			> 1200 g			*p*-value^a^			
	*n* = 25 gentamicin levels in 25 patients^b^			*n* = 24 gentamicin levels in 23 patients^b^			*n* = 15 gentamicin levels in 13 patients			Overall *p*-value	≤ 850 g vs 851 − 1200 g	≤ 850 g vs > 1200 g	851 − 1200 g vs > 1200 g
	Mean	95% Confidence Interval	Range	Mean	95% Confidence Interval	Range	Mean	95% Confidence Interval	Range				
Elimination rate constant (h^−1^)	0.06415	0.05762–0.07068	0.0456–0.1139	0.09087	0.08447–0.09728	0.0652–0.1327	0.09734	0.08519–0.10948	0.05693–0.14332	**< 0.0001**	**< 0.001**	**< 0.001**	> 0.05
Half-life (h)	10.8	9.8–11.8	6.1–15.2	7.6	7.1–8.2	5.2–10.6	7.1	6.0–8.2	4.8–12.2	–	–	–	–
Volume of distribution (L)	0.36	0.33–0. 39	0.22–0.51	0.51	0.46–0.57	0.33–0.87	0.88	0.76–1.00	0.53–1.23				–
Volume of distribution (L/kg)	0.55	0.50–0.60	0.35–0.83	0.50	0.46–0.54	0.38–0.76	0.52	0.43–0.61	0.26–0.96	0.2471	–	–	–
Clearance (L/h)	0.023	0.021–0.025	0.016–0.038	0.047	0.041–0.053	0.028–0.086	0.086	0.070–0.101	0.040–0.137	–	–	–	–
Clearance (L/h/kg)	0.035	0.032–0.038	0.026–0.056	0.045	0.041–0.049	0.032–0.067	0.050	0.043–0.058	0.028–0.081	**< 0.0001**	**< 0.01**	**< 0.001**	> 0.05

Multiple Comparison Test for data that did not pass the test for normality

^a^ANOVA with Tukey-Kramer Multiple Comparisons Test for data that passed the test for normality or a Kruskal-Wallis Test with Dunn’s Multiple Comparison Test for data that did not pass the test for normality

^b^One patient contributed 1 set of gentamicin levels to weight categories ≤ 850 g and 851-1200 g

Bold data indicates statistically significant *p*-values for a given parameter with either bivariate or multivariable analysis

### Monte Carlo simulation

MCS of weight-based dosing regimens were performed for neonates weighing ≤ 850 g (Table 5) and those weighing between 851 and 1200 g (Table 6). The MCS-identified optimal practical dosing regimens for conventional peaks (5–10 mg/L) and troughs (≤ 2 mg/L) were: 3.5 mg/kg given iv q48h in neonates weighing ≤ 850 g (probability of target peak and trough attainment of 86 and 100%, respectively) and q24h in neonates weighing 851 – 1200 g (probability of target peak and trough attainment of 91 and 97%, respectively). The MCS-identified optimal practical dosing regimens to produce higher peak concentrations of 12–20 mg/L and undetectable trough concentrations (≤ 0.5 mg/L) were: 8-9 mg/kg dose given iv q72h in neonates weighing ≤ 850 g (probability of target peak and trough attainment of > 73 and > 85%, respectively) and given q48h in neonates between 851 and 1200 g (probability of target peak and trough attainment of > 75 and > 84%, respectively).

**Table 5:** Monte Carlo Simulation Results for Neonates Weighing ≤ 850 g

Dosing Regimen		Target Peak Serum Concentration (mg/L)				Target Trough Serum Concentration (mg/L)		Peak:Minimum Inhibitory Concentration Ratio
Dose (mg/kg)	Dosing Interval (h)	5–10	12–20	15–20	≥ 20	≤ 2	≤ 0.5	≥ 8
2.5^a^	24	68.76%	0.12%	0.01%	0.00%	81.16%	5.18%	4.21%
3.5^a^	24	80.45%	4.61%	0.51%	0.01%	58.24%	1.68%	11.26%
3.0	48	69.05%	0.02%	0.00%	0.00%	99.89%	78.65%	3.26%
3.5^b^	48	86.31%	0.27%	0.00%	0.00%	99.74%	71.89%	6.35%
4.0	48	87.81%	1.53%	0.07%	0.00%	99.45%	65.51%	9.30%
4.5	48	78.22%	5.12%	0.40%	0.00%	99.01%	59.83%	12.54%
5.0	48	62.65%	12.44%	1.53%	0.02%	98.42%	53.62%	16.18%
5.5	48	45.62%	23.47%	4.10%	0.12%	97.69%	49.95%	20.08%
6.0	48	30.54%	36.89%	8.66%	0.42%	96.76%	45.74%	24.21%
6.5	48	18.92%	50.59%	15.39%	1.13%	95.72%	41.94%	28.74%
7.0	48	11.15%	62.11%	23.60%	2.61%	94.51%	38.77%	33.22%
7.5	48	6.10%	70.60%	32.27%	5.19%	93.14%	35.73%	37.96%
8.0	48	3.16%	74.94%	39.79%	9.10%	91.71%	32.96%	42.66%
8.0^c^	72	4.62%	73.37%	35.31%	5.94%	99.84%	87.65%	39.55%
8.5^c^	72	2.41%	76.38%	42.25%	10.04%	99.79%	86.31%	43.92%
9.0^c^	72	1.10%	75.99%	47.24%	15.56%	99.73%	84.88%	48.44%
9.5	72	0.45%	72.46%	49.45%	22.35%	99.65%	83.46%	52.81%
10	72	0.10%	66.97%	49.27%	30.06%	99.55%	82.07%	56.95%

^a^Dosing regimens recommended at Sunnybrook at time of study: ≤ 27 weeks corrected gestational age (CGA): 2.5 mg/kg q24h; 28–32 weeks CGA: 3.5 mg/kg q24h; 33–34 wks CGA: 4.5 mg/kg q24h

^b^Recommended dosing to target gentamicin concentrations: Peak 5-10 mg/L and Trough < 2 mg/L

^c^Recommended dosing to target gentamicin concentrations: Peak 12-20 mg/L and Trough ≤ 0.5 mg/L

**Table 6:** Monte Carlo Simulation Results for Neonates Weighing Between 851 and 1200 g

Dosing Regimen		Target Peak Serum Concentration (mg/L)				Target Trough Serum Concentration (mg/L)		Peak: Minimum Inhibitory Concentration Ratio
Dose (mg/kg)	Dosing Interval (h)	5–10	12–20	15–20	≥ 20	≤ 2	≤ 0.5	≥ 8
2.5^a^	24	66.21%	0.00%	0.00%	0.00%	99.66%	27.12%	3.27%
3.5^a^	24	91.17%	0.83%	0.02%	0.00%	96.57%	9.94%	9.39%
2.5	24	66.21%	0.00%	0.00%	0.00%	99.66%	27.12%	3.27%
3.0	24	90.28%	0.06%	0.00%	0.00%	98.68%	16.37%	6.08%
3.5^b^	24	91.17%	0.83%	0.02%	0.00%	96.57%	9.94%	9.39%
4.0	24	77.21%	4.42%	0.21%	0.00%	93.13%	6.17%	13.23%
4.5	24	55.29%	13.88%	1.23%	0.01%	88.39%	4.01%	17.50%
4.5	36	72.05%	5.81%	0.27%	0.00%	99.96%	69.44%	14.27%
5.0	36	50.61%	15.89%	1.38%	0.00%	99.90%	62.85%	18.27%
5.5	36	30.74%	31.43%	4.51%	0.05%	99.80%	56.50%	22.73%
6.0	36	16.46%	49.27%	10.87%	0.28%	99.66%	50.67%	27.44%
6.5	36	7.96%	65.32%	20.63%	0.96%	99.42%	45.56%	32.25%
7.0	36	3.50%	76.80%	32.38%	2.61%	99.08%	40.71%	37.26%
7.5	36	1.43%	82.50%	43.50%	5.85%	98.62%	36.70%	42.38%
8.0	36	0.56%	82.76%	52.04%	11.15%	98.06%	32.80%	47.42%
8.5	36	0.21%	78.31%	56.09%	18.62%	97.40%	29.56%	52.36%
9.0	36	0.07%	70.46%	55.41%	28.09%	94.61%	21.62%	66.33%
7.5	48	14.52%	81.31%	39.39%	4.12%	99.99%	89.88%	40.26%
8.0^c^	48	7.95%	83.70%	49.22%	8.35%	99.99%	88.06%	45.22%
8.5^c^	48	4.07%	81.27%	55.18%	14.72%	99.98%	86.11%	50.20%
9.0^c^	48	2.00%	74.96%	56.71%	23.04%	99.96%	84.10%	54.91%
9.5	48	0.94%	66.18%	54.11%	32.88%	99.95%	82.15%	59.48%
10	48	0.43%	56.10%	48.53%	43.50%	99.93%	80.12%	63.87%

^a^Dosing regimens recommended at Sunnybrook at time of study: ≤ 27 weeks corrected gestational age (CGA): 2.5 mg/kg q24h; 28–32 weeks CGA: 3.5 mg/kg q24h; 33–34 wks CGA: 4.5 mg/kg q24h

^b^Recommended dosing to target gentamicin concentrations: Peak 5-10 mg/L and Trough < 2 mg/L

^c^Recommended dosing to target gentamicin concentrations: Peak 12-20 mg/L and Trough ≤ 0.5 mg/L

## Discussion

This retrospective pharmacokinetic study evaluated hospitalized neonates with normal renal function, and a median CGA at gentamicin initiation of < 28 weeks. Seventy-five percent of those included were born at ≤ 28 weeks gestation and 92% had a BW of < 1500 g. Gentamicin Cl (L/h) and Vd (L) were significantly associated with weight at gentamicin initiation (≤ 850 g, 851-1200 g, and > 1200 g). Since no significant difference in pharmacokinetics existed for neonates weighing > 1200 g versus 851-1200 g, due to inadequate sample size in the largest weight category, we did not explore the > 1200 g sub-group further. No CART identified trees with breakpoints for the other MLR equation covariates (PNA and gender) existed. Based on the absence of CART identified trees and breakpoints for PNA and gender and the identification of a significant difference in both k_e_ and Cl (L/h/kg) between the ≤850 g and 851-1200 g sub-groups, the use of the simple weight range breakpoints of ≤850 g and 851-1200 g as the sub-groups for practical and convenient empiric gentamicin dosing calculations in neonates is rational. Dosing of 3.5 mg/kg/dose administered every 48 h for neonates weighing ≤ 850 g, and every 24 h for neonates weighing 851-1200 g provided the best probability of attaining conventional targets (peak:5-10 mg/L, trough:≤ 2 mg/L). Dosing of 8-9 mg/kg/dose administered every 72 h in neonates weighing ≤ 850 g and every 48 h in neonates weighing 851-1200 g provided the best probability of attaining EID targets (peak:12-20 mg/L, trough:≤ 0.5 mg/L).

The strengths of our study include the determination of gentamicin pharmacokinetics in a large sample of premature and low-birth weight neonates for whom data are currently lacking; the identification of significant covariates for Vd and Cl with determination of practical weight breakpoints; the utilization of MCS with 1 million iterations to develop simple initial gentamicin dosing nomograms for both conventional and EID for low-birth weight neonates with an excellent probability of target peak and trough attainment; and the provision of tables itemizing probabilities of target attainment (including Peak:MIC ratio) for a range of potential dosing options enabling institutional selection of initial dosing guidelines based on their GNB susceptibility patterns and desired target serum concentrations. In addition, our rigorous study design which limited the inclusion of gentamicin levels to those with a confirmed time for dose administration and serum sampling increases the validity of our results.

The weaknesses of our study include its retrospective design and associated risk of unrecognized confounders; the inability to generalize our results to neonates > 1200 g and SGA infants; and the risk of incomplete gentamicin distribution at time of sampling for peak concentrations. However, since our mean pharmacokinetic parameters were comparable to those reported in other studies [15], our sampling practice is unlikely to have affected the validity of our results.

Similar to other pharmacokinetic studies, our multivariable analysis indicated that the Vd of gentamicin in neonates is associated with body weight [15, 18–20, 28, 29]. Pharmacokinetic studies have identified that extracellular fluid volume correlates closely with bodyweight [35].

Our multivariable analysis indicated that gentamicin clearance in neonates is associated with PNA, as well as bodyweight, and gender. The correlation between PNA and gentamicin elimination has been previously reported in the literature [19, 28, 29], and is explained by the maturation of renal function in neonates. Since glomerulogenesis proceeds until 32–34 weeks gestation, preterm neonates are expected to have a reduced rate of glomerular filtration compared to their mature counterparts [36]. In the first 48–72 h of life there is a marked increase in glomerular filtration rate of full term newborns to rates of 8–20 ml/min, compared with increases in preterm neonates of only 2–3 ml/min [35, 37]. The half-life of elimination of gentamicin is therefore expected to decrease with increasing PNA because it is renally eliminated [37], as evidenced in our study. In addition, bodyweight likely serves as a surrogate marker for physiological maturity. Therefore, it is expected that the half-life of elimination of gentamicin decreases as body weight increases. This relationship was demonstrated in our study, as well as in previously published literature [15, 18–20, 29].

CART analysis confirmed breakpoints for weight at gentamicin initiation for both Vd and Cl and demonstrated that neonates had altered Vd (L) and Cl (L/h) based on these weight breakpoints. This allowed the use of the CART derived weight breakpoints (≤850 g and 851-1200 g) to divide our data into homogenous patient sub-groups for practical empiric gentamicin dosing recommendations and provides a new and convenient nomogram for gentamicin dosing (either conventional or EID) with a MCS demonstrated high probability of target attainment. The mean gentamicin Vd (0.55 L/kg and 0.50 L/kg for neonates weighing ≤ 850 g and 851-1200 g, respectively) and Cl (0.035 L/h/kg and 0.045 L/h/kg, for neonates weighing ≤ 850 g and 851-1200 g, respectively) identified in this study are comparable to those reported in a study of infants born at less than 28 weeks gestation (Vd = 0.50 L/kg and Cl = 0.032 L/h/kg) [15].

Our study confirms previous reports [2, 27, 38] that GNB, particularly *E coli*, are emerging as the leading cause of systemic infections in neonates. Recent microbiological reports of *E coli* isolates from Canadian pediatric patients report a mean MIC_90_ of 2 mg/L for gentamicin [33]. Therefore, to meet the PK/PD target of a peak: MIC ratio between 8 and 10, peak gentamicin concentrations should range from 16 to 20 mg/L. A single published study approximates these recommendations by targeting a peak concentration of 15–20 mg/L in neonates [19]. In this study, initial doses of 10 mg/kg administered at 36 h intervals were used in term newborns and 12 mg/kg doses administered every 48 h were used in premature neonates (GA 31–38 weeks) [19]. Our MCS derived initial EID recommendations for gentamicin of 8-9 mg/kg/dose administered every 72 h in neonates weighing ≤ 850 g and every 48 h in neonates weighing 851-1200 g has > 73% probability of attaining a peak between 12 and 20 mg/L and > 84% probability of attaining a trough of ≤ 0.5 mg/L. Our work is further supported by results from a recent study concluding that a prolonged dosing interval for gentamicin ranging from 36 to 72 h was appropriate for neonates weighing less than 1000 g [25]. However, our results provide a new easy to use gentamicin dosing nomogram for both conventional and EID gentamicin with a MCS demonstrated high probability of target attainment, which has not previously been completed for neonates. In all cases the weight based initial dosing recommendations derived in our study provided a better probability of target attainment than the CGA-based gentamicin dosing regimens used at our institution at the time of this study conduct. In 2014 our centre changed its gentamicin dosing practice to adopt the weight based nomogram developed in this study; where EID is now predominantly used for NICU babies. We have received positive feedback about the simplicity, safety and efficacy of the nomogram from our NICU physicians and pharmacists. Plans are underway to evaluate the safety, efficacy and health care personnel workload of the weight based nomograms for conventional and EID using a pragmatic study design.

Although the study by Lanao et al [19] was published in 2004, higher peak concentration targets have not been routinely adopted by clinicians. Therefore, we chose to report the probabilities of achieving a range of peak gentamicin concentrations with various dosing regimens because GNB MICs, along with desired target peak concentrations, may vary among hospitals. Our MCS dosing tables may assist clinicians in choosing a gentamicin dosing regimen that would be optimal based on their institutional MIC patterns for relevant GNB, such as *E. coli*.

## Conclusions

The study contributes new data based gentamicin dosing guidelines for both initial conventional and EID in neonates ≤ 1200 g, a patient population under-represented in neonatal studies and for whom limited data exists for gentamicin dosing. Our results provide clinicians with practical and simple initial dosing recommendations based on weight at time of gentamicin initiation with a high probability of target peak and trough attainment. Confirmatory gentamicin levels (peak and trough with third dose for conventional therapy and a peak and 8–12 h post level with the first dose of EID) are recommended to further refine dosing. If more prolonged therapy is needed, then repeat levels are recommended to identify changes in the neonate’s gentamicin pharmacokinetics with PNA and weight. The gentamicin levels that were targeted in this study reflect accepted safe and effective levels for gentamicin in neonates [1, 2, 6, 11–26]. However, due to the retrospective design of our study, a prospective pharmacokinetic clinical study in neonates ≤ 1200 g is needed to confirm the efficacy and safety of the gentamicin EID nomogram recommendations.

## Data Availability

The datasets used and/or analysed during the current study are available from the corresponding author on reasonable request.

## Appendix 1

**Table 7:** Sunnybrook Health Sciences Centre’s Dosing Recommendations for Gentamicin in Neonates at Time of Study (2013)

Corrected Gestational Age [Weeks]	Dose [mg/kg]	Dosing Interval [Hours]	Administration Technique	Serum Concentration Sampling
< 28	2.5	24	1 h infusion	Immediately following end of infusion
28–32	3.5	24	1 h infusion	Immediately following end of infusion
33–34	4	24	1 h infusion	Immediately following end of infusion
35–36	2	12	Intravenous bolus	30 min following bolus
> 36	2.5	12	Intravenous bolus	30 min following bolus

## Appendix 2

**First Order Pharmacokinetic Equations:**

1. $$ \mathrm{k}=\frac{\hbox{-} \left(\ln\ {\mathrm{C}}^{\mathrm{obs}.\min}\hbox{-} \ln\ {\mathrm{C}}^{\mathrm{obs}.\max}\right)}{\mathrm{t}\mathrm{min}-\mathrm{tmax}\kern0.75em };{\mathrm{t}}_{1/2}=0.693/\mathrm{k} $$
2. $$ {\mathrm{C}}^{\operatorname{ext}.\max }=\frac{{\mathrm{C}}^{\mathrm{obs}.\max }}{{\mathrm{e}}^{\hbox{-} \mathrm{ktmax}}} $$
3. $$ {\mathrm{C}}^{\operatorname{ext}.\min }={\mathrm{C}}^{\operatorname{ext}.\max}\mathrm{x}\ {\mathrm{e}}^{\hbox{-} \mathrm{k}\left(\mathrm{t}\hbox{-} \mathrm{t}\hbox{'}\right)} $$
4. $$ \mathrm{V}=\frac{\mathrm{Dose}\ \mathrm{x}\ \left[1\hbox{-} {\mathrm{e}}^{\hbox{-} \mathrm{kt}\hbox{'}}\right]}{\mathrm{kt}\hbox{'}\kern0.5em \left[{\mathrm{C}}^{\operatorname{ext}.\max}\hbox{-} {\mathrm{C}}^{\operatorname{ext}.\min}\mathrm{x}\ {\mathrm{e}}^{\hbox{-} \mathrm{kt}\hbox{'}}\right]} $$
5. Suggested Dosing Interval:

$$ \mathrm{t}=\frac{\hbox{-} 1/\mathrm{k}\ \ln \left({\mathrm{C}}^{\mathrm{des}.\mathrm{minss}}\right)+\mathrm{t}\hbox{'}}{\left({\mathrm{C}}^{\mathrm{des}.\mathrm{maxss}}\right)} $$

Round to 4, 6, 8, 12, 16, 18, 24 etc.

6. Suggested Maintenance Dose:

$$ \mathrm{Dose}={\mathrm{kVC}}^{\mathrm{des}.\mathrm{maxss}}\mathrm{x}\frac{\left(1\hbox{-} {\mathrm{e}}^{\hbox{-} \mathrm{kt}}\right)\kern0.5em \mathrm{x}\ \mathrm{t}\hbox{'}}{\left(1\hbox{-} {\mathrm{e}}^{\hbox{-} \mathrm{kt}\hbox{'}}\right)} $$

7. Predicted Peak

$$ {\mathrm{C}}^{\mathrm{pred}.\mathrm{maxss}}=\frac{\mathrm{Dose}\ \mathrm{x}\ \left(1\hbox{-} {\mathrm{e}}^{\hbox{-} \mathrm{kt}\hbox{'}}\right)}{\mathrm{t}\hbox{'}\mathrm{kV}\ \mathrm{x}\ \left(1\hbox{-} {\mathrm{e}}^{\hbox{-} \mathrm{kt}}\right)} $$

8. Predicted Trough

$$ {\mathrm{C}}^{\mathrm{pred}.\mathrm{minss}}={\mathrm{C}}^{\mathrm{pred}.\mathrm{maxss}}\left({\mathrm{e}}^{\hbox{-} \mathrm{k}\left(\mathrm{t}\hbox{-} \mathrm{t}\hbox{'}\right)}\right) $$

**Key:**

- k= elimination rate constant (h^-1^) ; t_1/2_ = half-life (h)
- Cobs^.min^ & C^obs.max^ = observed minimum and maximum concentrations
- tmin & tmax = time post infusion (in hours) of observed minimum and maximum concentrations
- C^ext.min^ & C^ext.max^ = extrapolated or actual minimum and maximum concentrations
- V=volume of distribution (L); t = dosing interval in hours ; t' = infusion time in hours
- C^des.minss^ & C^des.maxss^ = desired minimum and maximum concentrations at steady state
- C^pred.minss^ & C^pred.maxss^ = predicted minimum and maximum concentrations at steady state

## Abbreviations

ANOVA: Analysis of variance
BUN: Blood urea nitrogen
BW: Birth weight
CART: Classification and regression tree
CGA: Corrected gestational age
CI: Confidence interval
Cl: Clearance
EID: Extended-interval dosing
GA: Gestational age
GNB: Gram negative bacteria
ke: Elimination rate constant
MCS: Monte Carlo Simulation
MIC: Minimum inhibitory concentration
MLR: Multiple linear regression
NICU: Neonatal intensive care unit
PK: Pharmacokinetic
PNA: Post-natal age
sCr: Serum creatinine
SD: Standard deviation
SGA: Small for gestational age
SHSC: Sunnybrook Health Sciences Centre
t_1/2_: Half-life
TDM: Therapeutic drug monitoring
Vd: Volume of distribution
